# Supplementary material for: Serendipita indica E5′NT modulates extracellular nucleotide levels in the plant apoplast and affects fungal colonization
Source: EMBO Rep. 2019 Feb 5;20(2):e47430. doi: 10.15252/embr.201847430 (PMC6362346; doi:10.15252/embr.201847430)
Supplement: Supplementary file 1 — Appendix [file EMBR-20-e47430-s001.pdf]

Appendix

Serendipita indica E5′NT modulates extracellular nucleotide levels in the plant apoplast and affects fungal colonization

Authors

Shadab Nizam[1,2], Xiaoyu Qiang[1,2], Stephan Wawra[2], Robin Nostadt[1], Felix Getzke[2], Florian Schwanke[2], Ingo Dreyer[3], Gregor Langen[2], Alga Zuccaro[1,2+]

Table of contents

Appendix Figures	Page
Appendix Figure S1	S2
Appendix Figure S2	S3
Appendix Figure S3	S4
Appendix Figure S4	S5
Appendix Figure S5	S6
Appendix Figure S6	S7
Appendix Figure S7	S8
Appendix Figure S8	S9
Appendix Figure S9	S10-11
Appendix Figure S10	S12
Appendix Figure S11	S13
Appendix Figure S12	S14
Appendix Figure S13	S15
Appendix Figure S14	S16

Appendix Figure S1

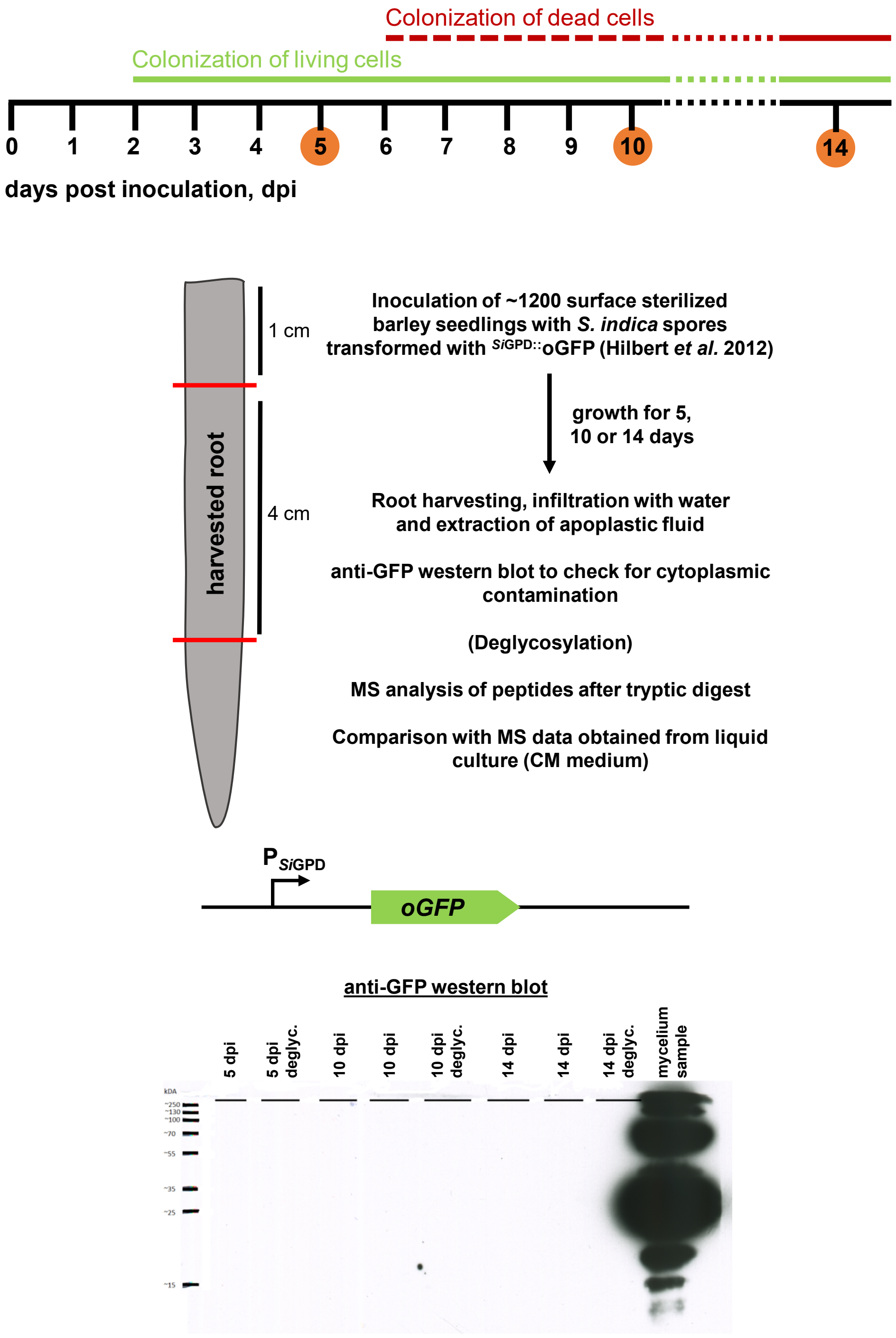

Appendix Figure S1: Schematic workflow describing the barley inoculation and apoplastic fluid (APF) collection. To ensure that no apoplastic leakage of the *S. indica* GoGFP strain expressing cytosolic GFP used for the inoculation of the barley seedling occurred during APF collection the samples dedicated for mass spectrometric analysis were examined by an anti-GFP western blot.

Appendix Figure S2

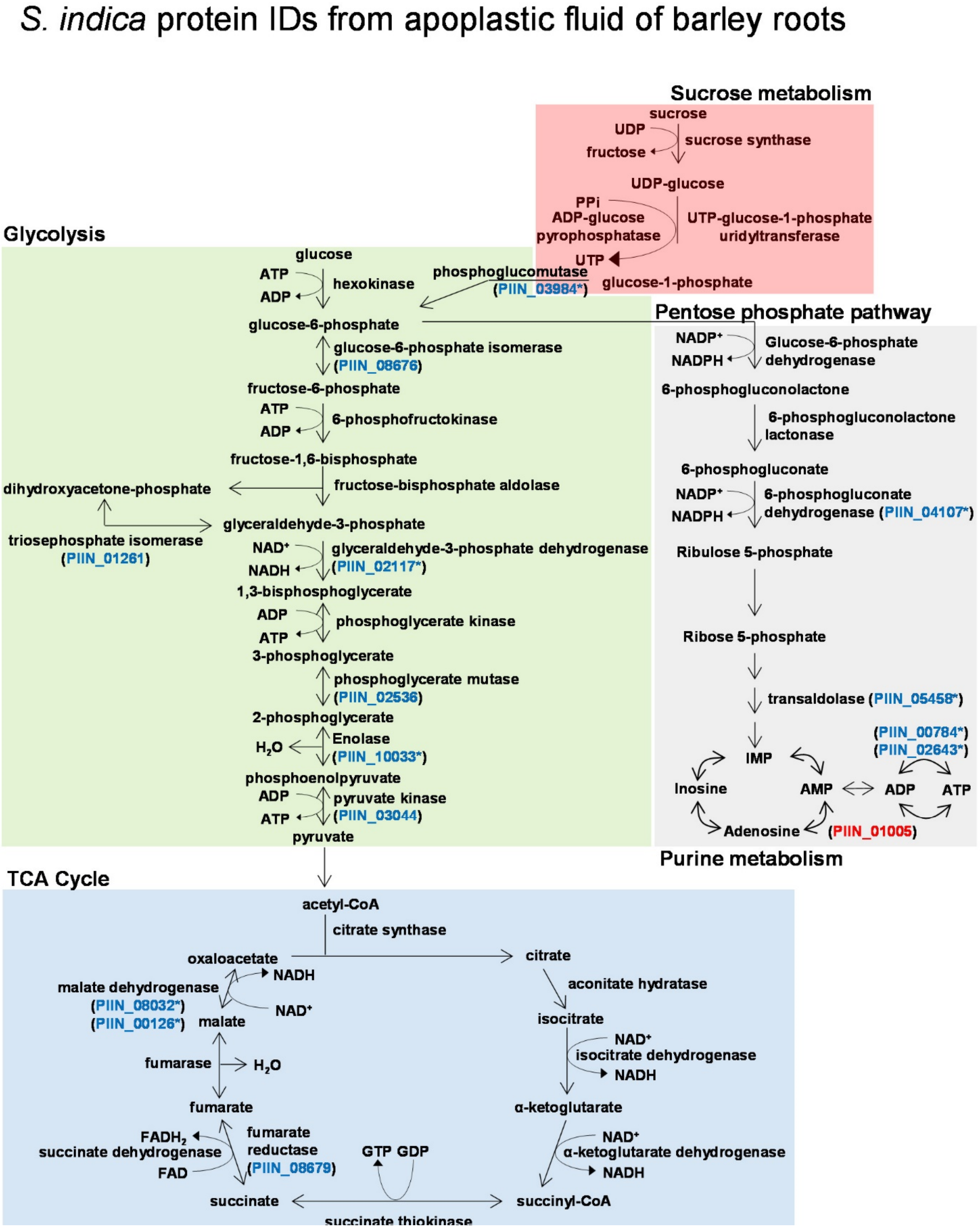

Appendix Figure S2: *S. indica* putative apoplastic proteins involved in metabolism shown within their respective cellular pathways. Asterisks indicate orthologues identified in the APF of rice leaves infected with the rice-blast pathogen *Magnaporthe oryzae* [43]. The metabolic pathways were modified from [77].

Appendix Figure S3

Analysis of 102 APF proteins: Enriched GO terms

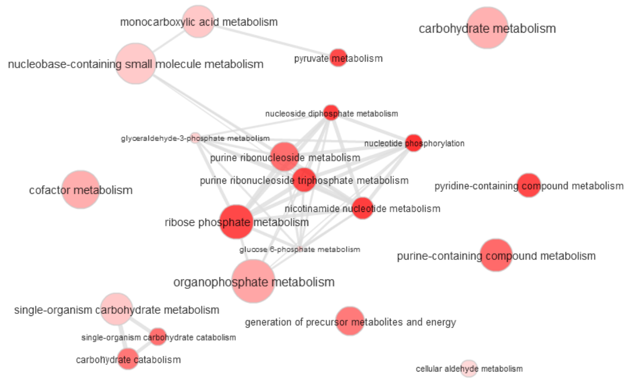

Analysis of 57 CF proteins: Enriched GO terms

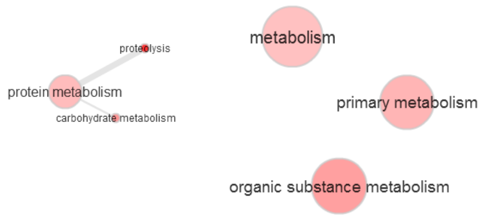

REVIGO visualisation of GOeast analyses results.
Bubble color indicates the p-value calculated by GOeast; bubble size indicates the frequency of the GO term. Highly similar GO terms are linked by edges in the graph, where the line width indicates the degree of similarity.
http://omicslab.genetics.ac.cn/GOEAST/ ; http://revigo.irb.hr/revigo.jsp

Appendix Figure S3: Gene Ontology Enrichment Analyses of apoplastic and culture filtrate proteins visualized using REVIGO. Detailed GOeast results are listed in Table EV3.

Appendix Figure S4

Peptides for E5'NT identified by LC-MS/MS from APF of barley roots:

HQLGIIGVITPDTK
GTSSGAGPGTEFSDPVQAVQK
QLLDIFEGVVSK
MNIAFDASGK

PIIN_01005

MQLSRSFALLAIYTTLVLAKVDSLVSERALQKRFVDANGNYNVTIVHTNDVHAHLDQWR
AGRGTDCTPGSECISGYARIKQKVSELRQSIQDPIFLNAGDEFQGTLFFTYYGGEKISYA
INEVGYDVFTLGNHEFDRGQGELAAFLKNLTFPVVCANFKTNDTAMNALNIQPYTIIEKH
QLGIIGVITPDTKGTSSGAGPGTEFSDPVQAVQKAVDELQAKNITRIIALTHIGYDKDIELA
QKTKGVDLIVGGHSHTLLGNFTNAMGSYPTTAKNLDGEEVFIVTSYRWGEILGKMNIAF
DASGKIVSYEGEPLRLTNTTQQDPKLQAEVNEWRQPFDAMAQVVVGTSSVVLDQSIC
QFSECTLGNVITDAMYEYRKNAGGNVDFALINSGGIRASISEGTVTQGDILTSFPFMNG
VVDLTWTGKQLLDIFEGVVSKYSTLSHHATTSFIQVSKQVKFSWNPNNVNQTRLITLEIG
GQQVDVNKNYTMVTLDFLASNGDYLWGTRTDFAALDTLDVVLASYFKAHSPVGASIEG
RITNSTSTTQQLPGKGSSGGSGGASALIVSGLSAGMMLLAGMMLV

Peptide searches against barley proteome retrieved no perfect matches:

HQLGIIGVITPDTK
Best blastP hit vs Hordeum vulgare (taxid:4513)_ Sequence ID: CAX51385.1:
```
Query  4    GIIGVITPD  12
            G++GVITPD
Sbjct  223  GVVGVITPD  231
```

GTSSGAGPGTEFSDPVQAVQK
Best blastP hit vs Hordeum vulgare (taxid:4513)_ Sequence ID: BAJ89828.1:
```
Query  9    GTEFSDPVQAV  19
            GT+F DPV AV
Sbjct  205  GTDFPDPVAAV  215
```

QLLDIFEGVVSK
Best blastP hit vs Hordeum vulgare (taxid:4513)_ Sequence ID: BAJ94588.1:
```
Query  1    QLLDIFEGVV-SK  12
            QLLD FE +V SK
Sbjct  673  QLLDSFEEIVASK  685
```

MNIAFDASGK
Best blastP hit vs Hordeum vulgare (taxid:4513)_ Sequence ID: BAK02710.1:
```
Query  2    NIAFDASG  9
            NI FDA G
Sbjct  169  NISFDANG  176
```

Appendix Figure S4: Peptides identified by LC-MS/MS for E5′NT found in the APF of barley. BLAST searches against barley databank retrieved no perfect matches, indicating that these peptides originated from *S. indica* E5′NT.

Appendix Figure S5

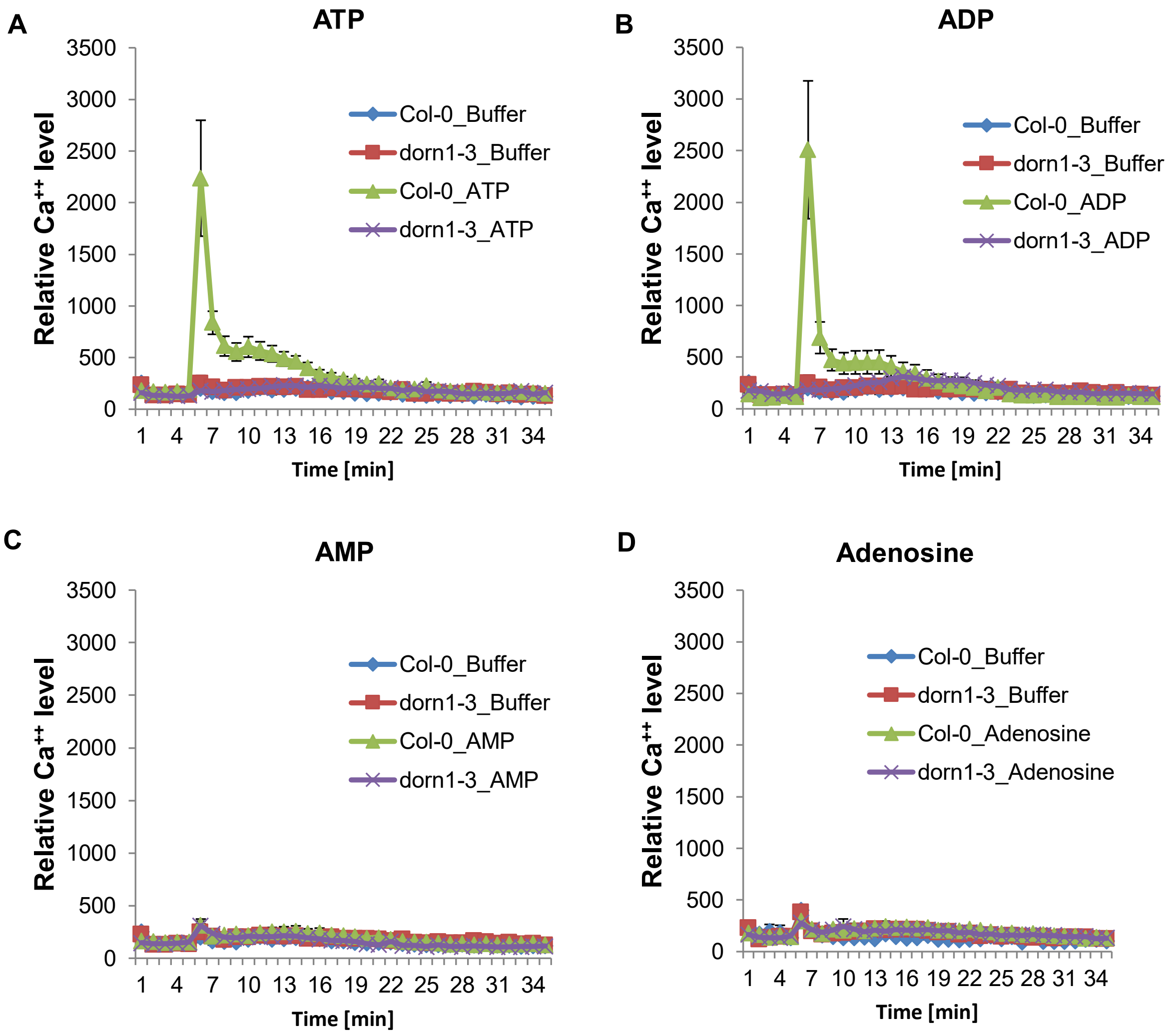

Appendix Figure S5: Characterization of the *dorn1-3* mutant line. ATP and ADP but not AMP nor adenosine induce intracellular calcium release in *Arabidopsis thaliana* expressing aequorin lines (Col-0^{aq}). The ATP receptor deletion mutant *dorn1-3* does not respond to ATP and ADP. After 5 min of background measurements 50 µl of 100 µM adenylates were added, subsequently luminescence was monitored for 30 min. **A**, ATP; **B**, ADP; **C**, AMP; **D**, adenosine. Data points represent means of six independent biological replicates ±SE.

Appendix Figure S6

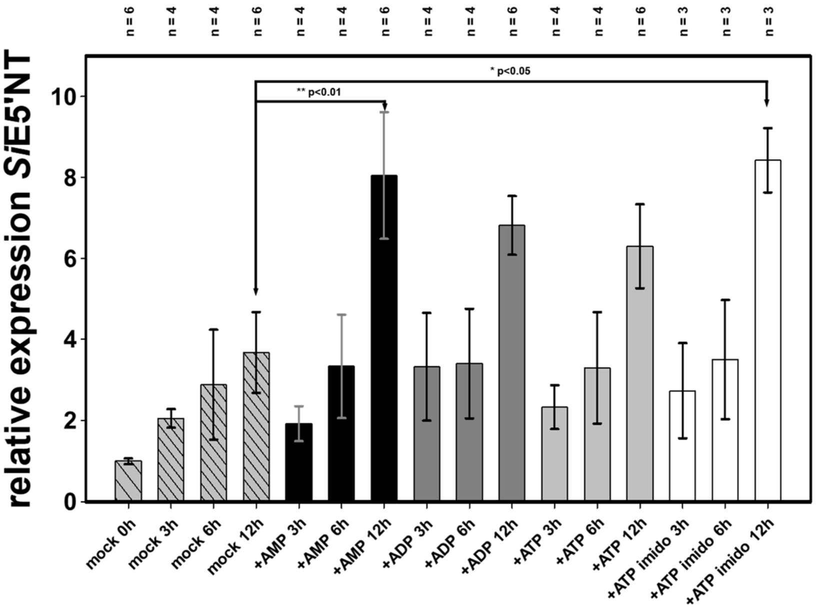

Appendix Figure S6: Expression of *Si*E5′NT in *S. indica* grown in liquid CM supplemented with 100 µM ATP, ADP, AMP or ATP-imido [adenosine 5′-(β,γ-imido)triphosphate, SIGMA] a non-hydrolysable form of ATP. Mock 0 represents the time point 0 just before addition of the nucleotides and it is set to 1. Y axis shows the relative expression of *Si*E5'NT. An induction of expression was detected at 12 hour post treatment for AMP and ATP-imido. Significances obtained by one way ANOVA testing of each individual time point are indicated. Error bars show the standard error of the mean from 4 biological replicates.

Appendix Figure S7

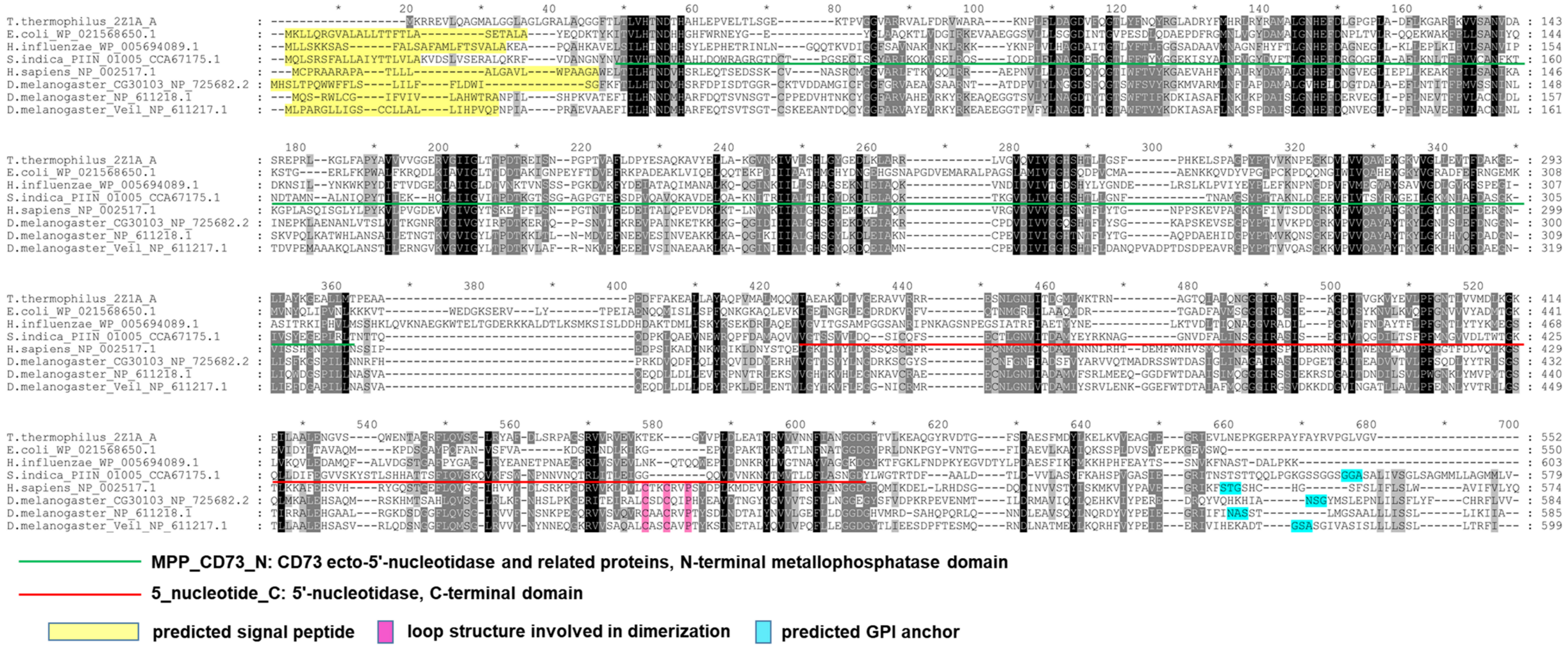

Appendix Figure S7: Sequence alignment of *S. indica,* bacterial and animal 5′NTs. *Si*E5′NT and animal E5′NT contain GPI anchors (predicted by FragAnchor; turquoise) and animal E5′NT a loop involved in dimerization (pink).

Appendix Figure S8

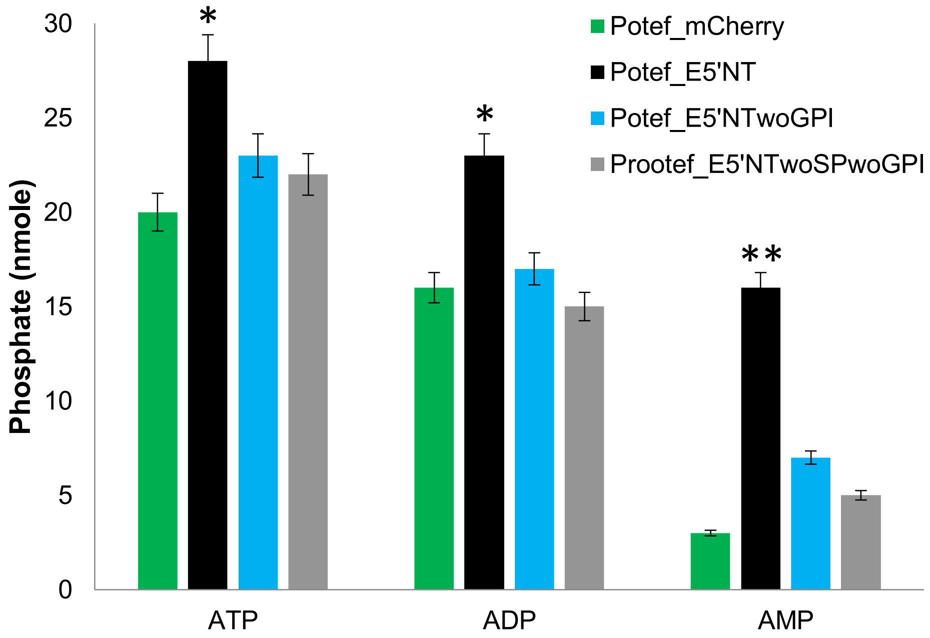

Appendix Figure S8: Characterization of *Si*E5′NT. A Cell surface 5′-nucleotidase activity assay of *Ustilago maydis* strains expressing different forms of *Si*E5′NT. To study the ecto-5′-nucleotidase activity of *Si*E5′NT and its substrate preferences, the biotrophic plant pathogen smut fungus *U. maydis* was chosen since an E5′NT orthologue predicted to be secreted is absent in its genome. Ecto-5′-nucleotidase activity of *U. maydis* cells expressing different *Si*E5′NT versions under the control of the constitutive synthetic otef promoter [64] were analyzed. ^{Potef}::mCherry encodes for cytosolic mCherry, ^{Potef}::E5′NT encodes for the full length *S. indica* enzyme, the ^{Potef}::E5′NTwoGPI construct lacks the predicted GPI anchor sequence and ^{Potef}::E5′NTwoSPwoGPI is missing both the predicted signal peptide and the GPI anchor. Cell surface activity of *Si*E5′NT in *U. maydis* washed cell suspensions were analyzed by measuring the amount of phosphate released after 30 min of incubation with 50 μM of either ATP, ADP or AMP. The amount of phosphate is significantly increased in cells transformed with ^{Potef}::E5′NT for all substrates tested. Error bars represent standard deviation of the mean from three biological replicates. Asterisks indicate significance at $p < 0.05$ (*), 0.01 (**) analyzed by Student's t-test to the mCherry control strains.

Appendix Figure S9

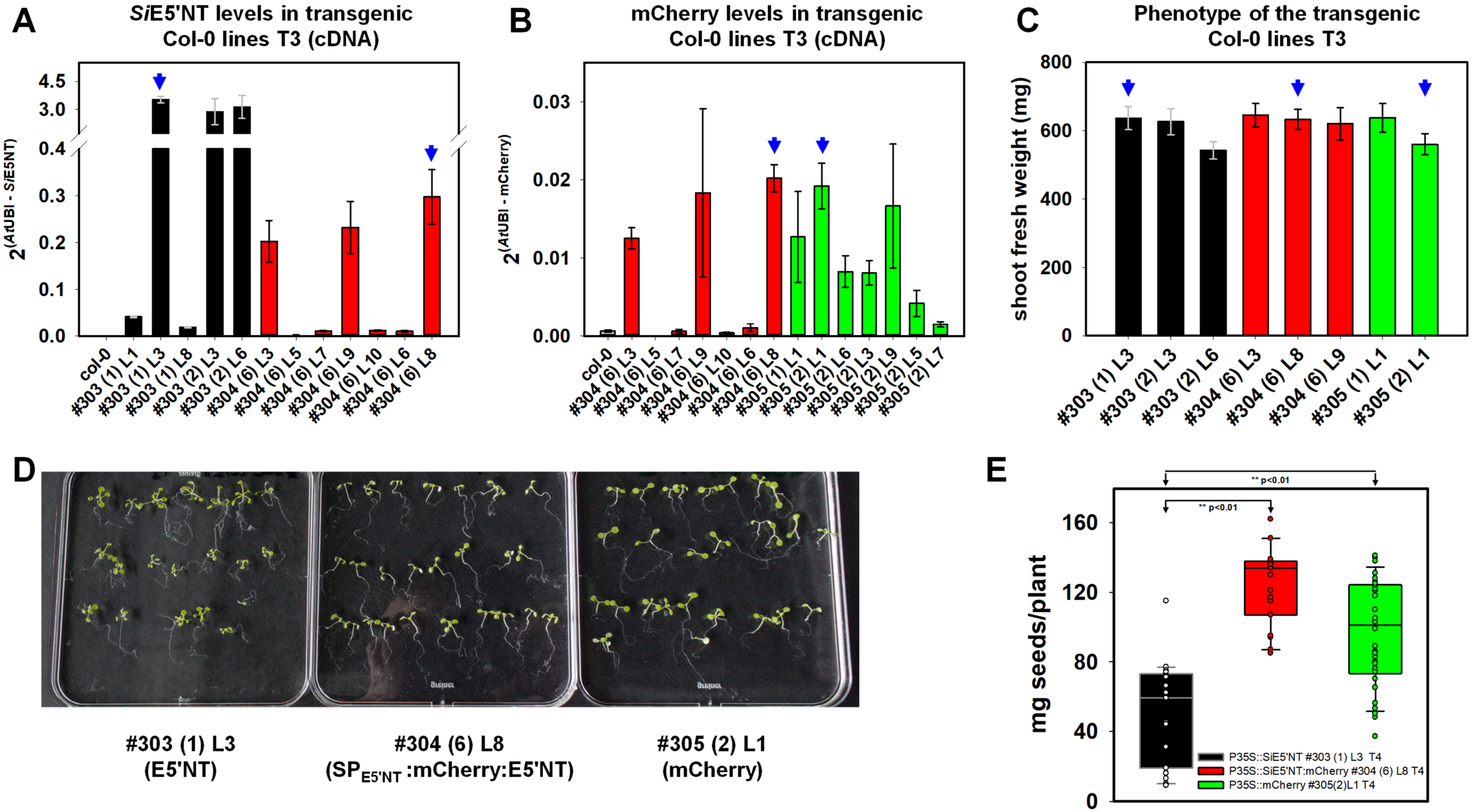

Supplementary figure 9: Characterization of *A. thaliana* T3 and T4 transgenic lines in Col-0 background. A *Si*E5′NT and **B** mCherry expression analysis of different *A. thaliana* T3 transgenic lines by qPCR. Error bars represent standard deviation of the mean from three technical replicates of one biological experiment. **C** Shoot weight of *A. thaliana* transgenic T3 lines. Error bars represent standard deviation of the mean from in total 12 plants of

two independent biological experiments. Arrows indicate the lines that were selected for further analyses. Black indicates E5′NT expressing lines; Red indicates E5′NT:mCherry expressing lines; Green indicates mCherry expressing lines. **D** Images of seedlings grown on ½ MS with Hygromycin B (15 µg/mL) from the T3 transgenic lines used for detailed analyses. **E** Seed production in the *A. thaliana* transgenic T4 lines used for detailed analyses in Figures S10 and S14. Box plots show the quartile of seed weights of the plant individuals analyzed. The overlaid dot plots shows the respective individual values. Plants were propagated in parallel in greenhouse in soil. Seeds from each plants were collected separately and weighted. Twenty to forty plants for each line were used for one way ANOVA statistical analysis. The activity of *Si*E5′NT might affects seed production in the #303 (1)L3 line by manipulation of eATP levels during propagation. It was reported that optimum concentration of eATP is essential for initiation of pollen germination in *A. thaliana* and in Gymnosperms [78, 79].

Appendix Figure S10

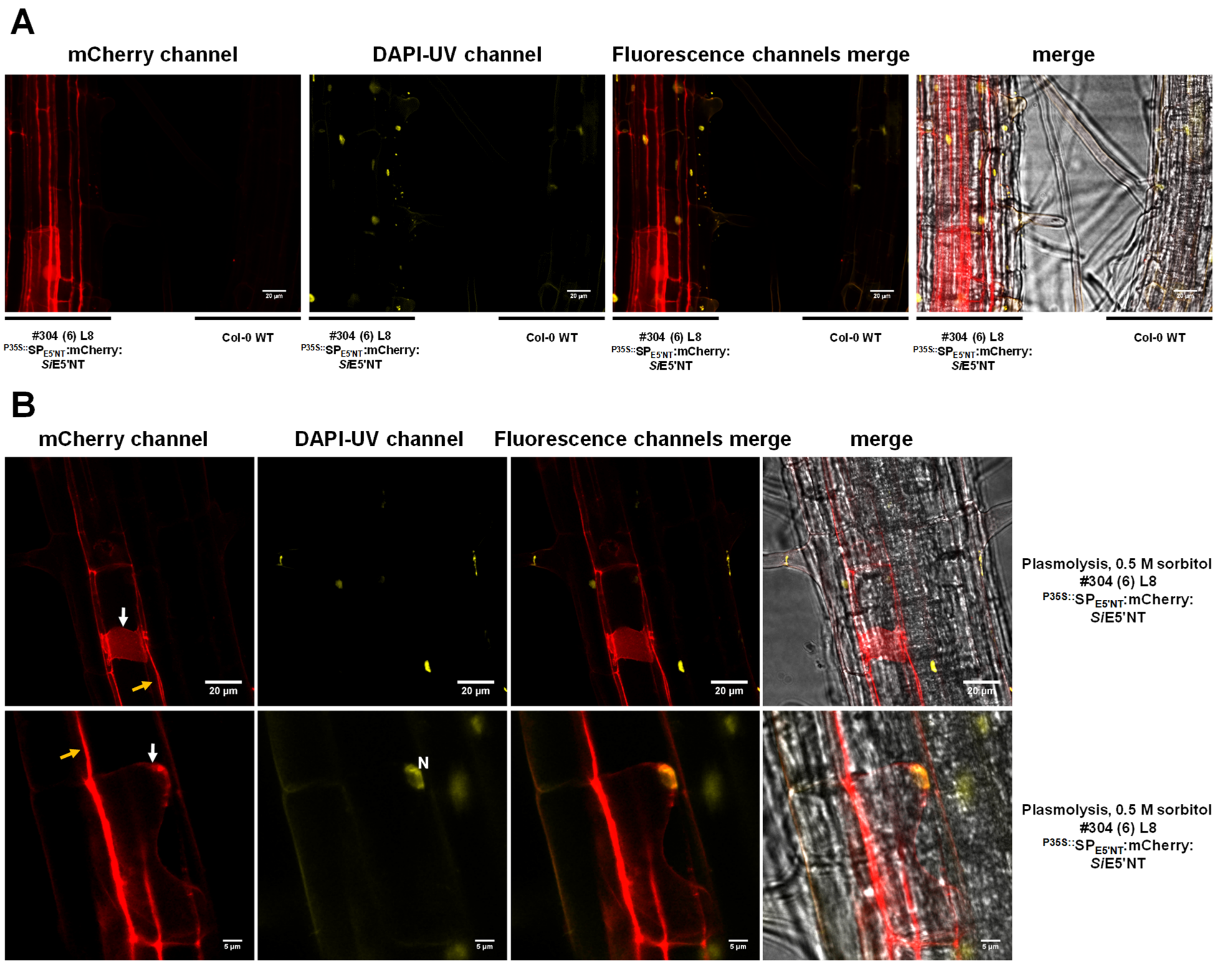

Appendix Figure S10: A Confocal laser scanning microscopy of roots from the lines #304 (6)L8 expressing E5′NT:mCherry and Col-0 WT under the same settings. Plant nuclei were stained with DAPI and visualized with the UV channel. No auto-fluorescence in the UV channel was observed at the laser intensity used. No fluorescence signal was observed in the WT root in the mCherry (red) channel. **B** Plasmolysis using 0.5 M sorbitol. After plasmolysis a fluorescence signal was detected at the membrane (white arrows) and at the cell wall (yellow arrows), suggesting that E5′NT:mCherry fusion protein is secreted. N indicates a nucleus.

Appendix Figure S11

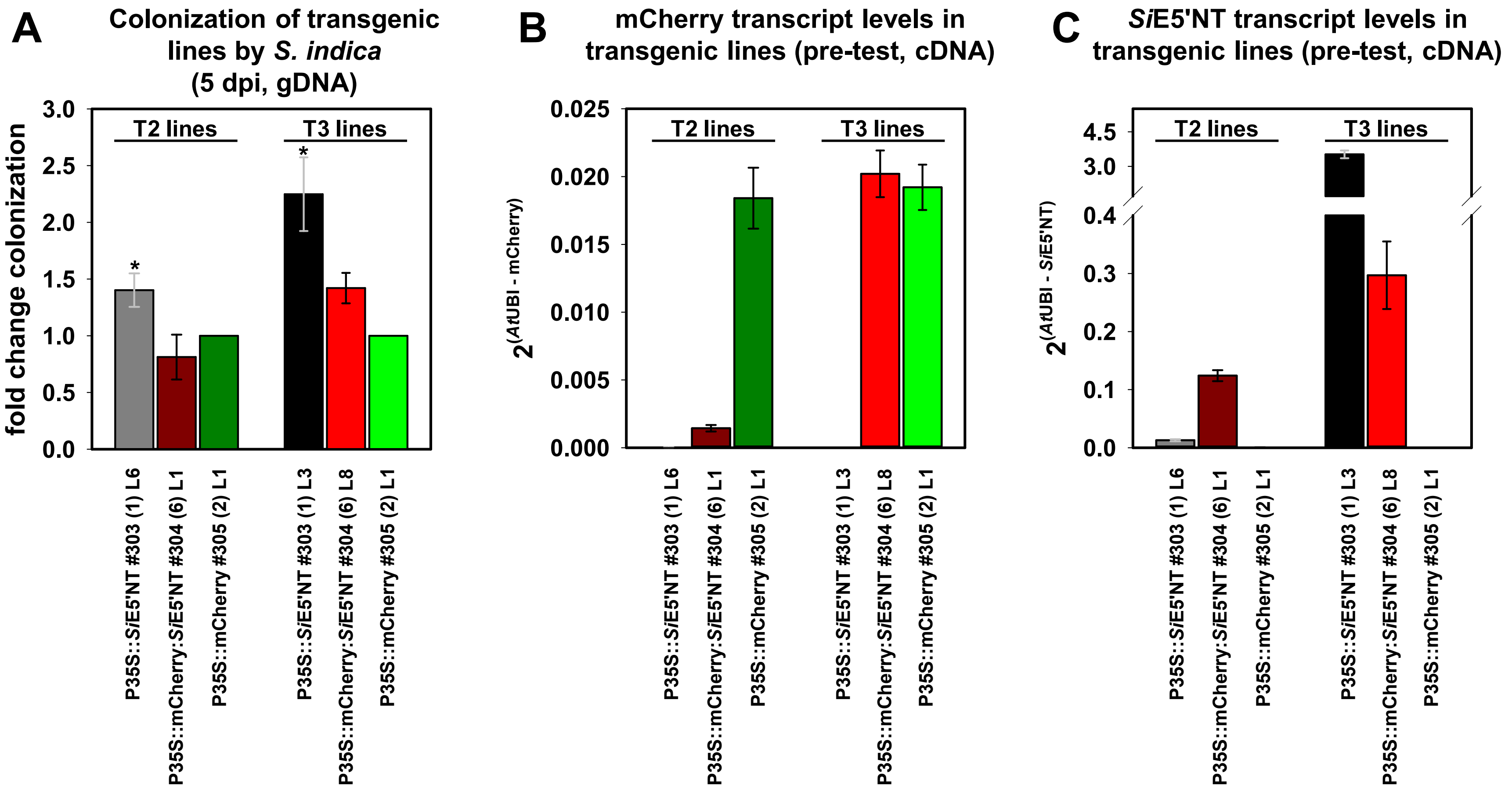

Appendix Figure S11: A *S. indica* colonization levels of different independent transgenic T2 and T3 lines. B mCherry transcript levels of the respective untreated plant lines. **C** *Si*E5′NT transcript levels of the respective untreated plant lines. Samples were analyzed by qPCR. Error bars show the standard error of the mean of three independent biological replicates. Asterisks indicate significance at p< 0.05 (*) analyzed by Student's t-test to the mCherry control line (#305).

Appendix Figure S12

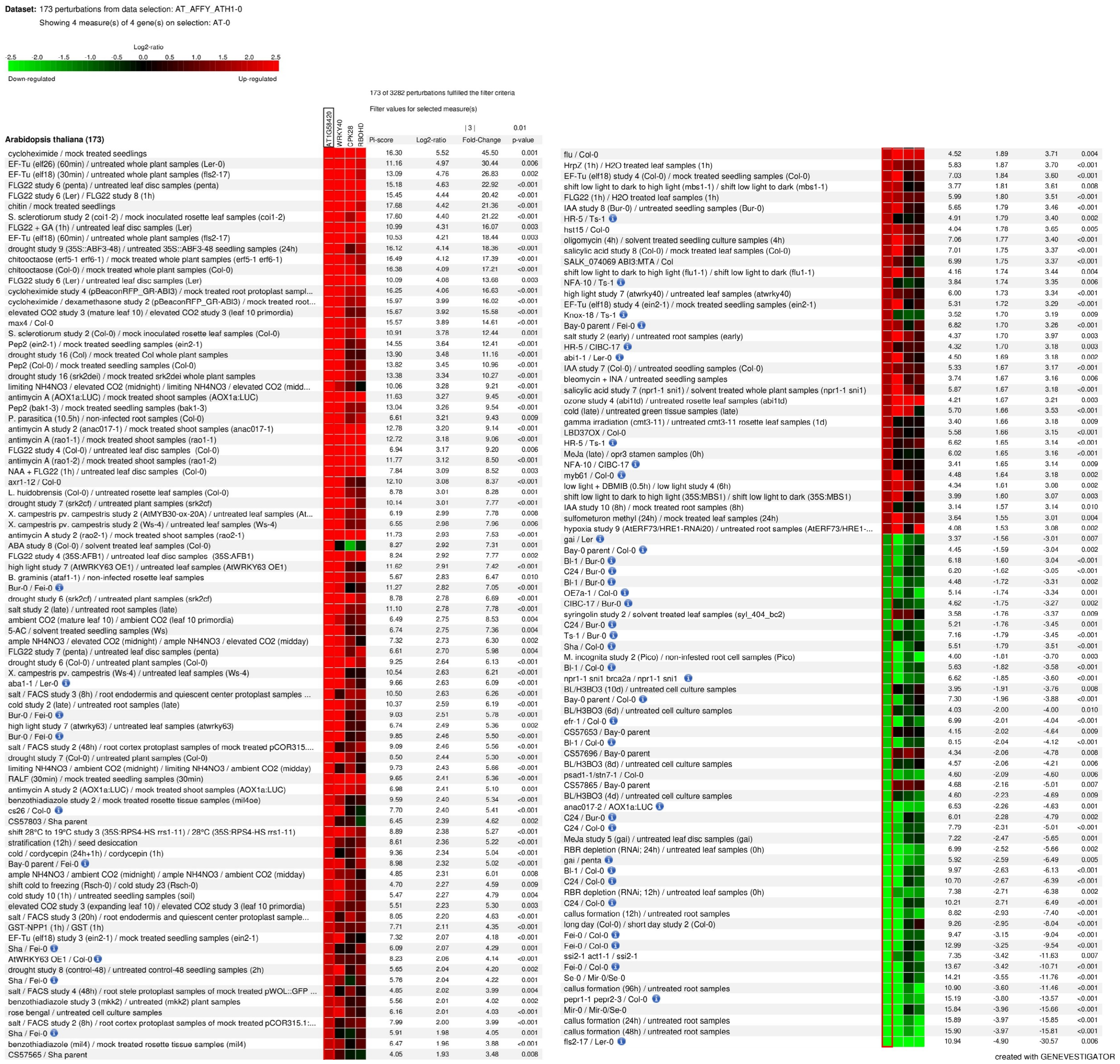

Appendix Figure S12: Expression patterns of At1g58420, *At*WRKY40, *At*RBOHD and *At*CPK28 under different perturbations, available in the GENEVESTIGATOR database. The filter for the generation of the dataset was set to a cutoff of fold change >3. These genes are eATP responsive only at low level (below cutoff) and strongly responsive to MAMPs and biotic stresses.

Appendix Figure S13

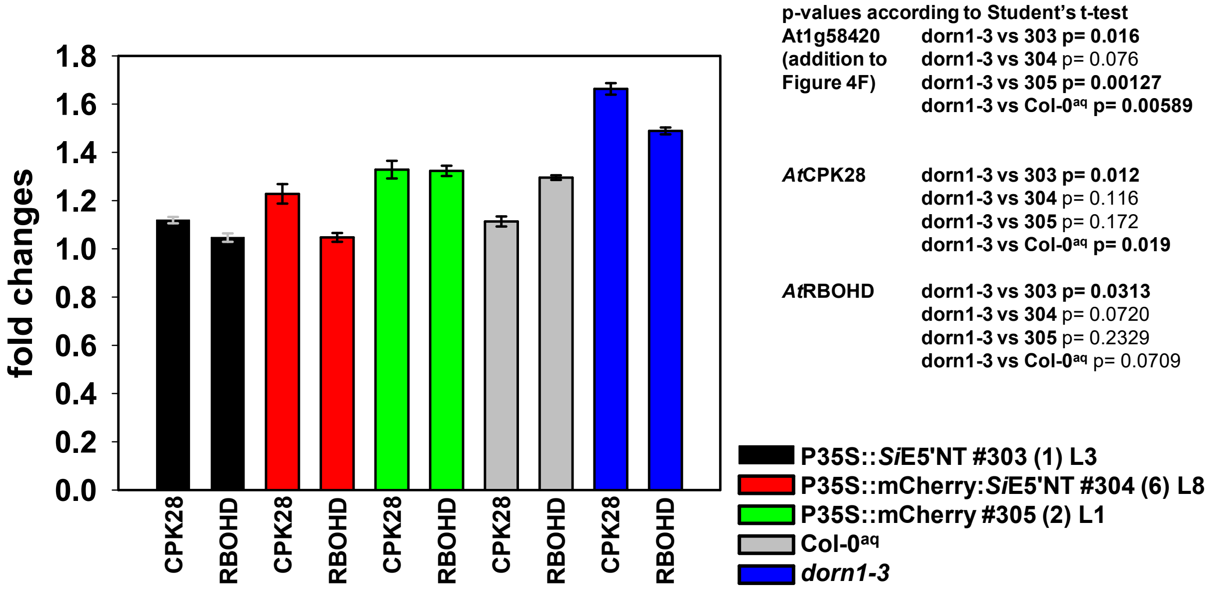

Appendix Figure S13: *At*RBOHD and *At*CPK28 expression levels upon *S. indica* colonization (addition to Figure 4E/F). Expression levels of the eATP and wounding responsive genes *At*RBOHD and *At*CPK28 [8] in transgenic lines colonized by *S. indica* at 5 dpi. For visualization data were normalized by setting the values from the mock treated samples to 1. Error bars represent ±SE of the mean from three independent biological replicates.

Appendix Figure S14

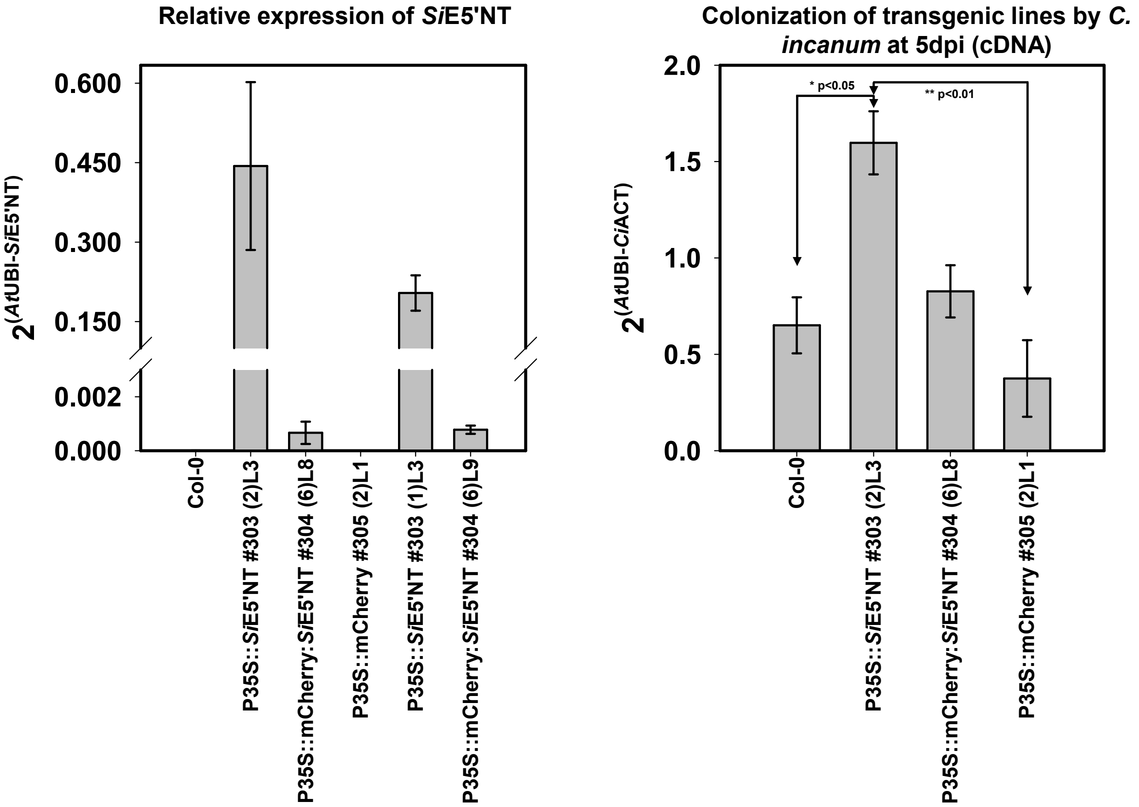

Appendix Figure S14: Colonization assay of transgenic lines by the phytopathogenic fungus *Colletotrichum incanum*. Left: Characterization of the transgenic lines after propagation in the T4 generation. Plants were propagated in parallel in greenhouse in soil. Lines #303 (2)L3, #304 (6)L8 and #305 (2)L1 were chosen for colonization analysis with *C. incanum* based on the expression strength for the respective heterologous gene. *Si*E5′NT transcript levels were measured by qPCR. **Right:** Root colonization of transgenic lines by *C. incanum* at 5 dpi. Error bars show the standard error of the mean of three independent biological replicates. Asterisks indicate significance at p< 0.05 (*) and p< 0.01 (**) analyzed by one way ANOVA.
